# Baricitinib and Lonafarnib Synergistically Target Progerin and Inflammation, Improving Lifespan and Health in Progeria Mice

**DOI:** 10.3390/ijms26104849

**Published:** 2025-05-19

**Authors:** Peter Krüger, Moritz Schroll, Felix Quirin Fenzl, Ramona Hartinger, Eva-Maria Lederer, Agnes Görlach, Leslie B. Gordon, Paola Cavalcante, Nicola Iacomino, Birgit Rathkolb, Juan Antonio Aguilar Pimentel, Manuela Östereicher, Nadine Spielmann, Cordula Maria Wolf, Martin Hrabe de Angelis, Karima Djabali

**Affiliations:** 1 Epigenetics of Aging, Department of Dermatology and Allergy, TUM School of Medicine and Health, Munich Institute of Biomedical Engineering (MIBE), Technical University of Munich (TUM), 85748 Garching, Germany; peter.krueger@tum.de (P.K.);; 2 Experimental and Molecular Pediatric Cardiology, Department of Pediatric Cardiology and Congenital Heart Diseases, German Heart Center Munich, Technical University Hospital, TUM School of Medicine and Health, 80636 Munich, Germany; 3 German Centre for Cardiovascular Research (DZHK), Partner Site Munich Heart Alliance, 80636 Munich, Germany; 4 Department of Anesthesia, Boston Children’s Hospital, Harvard Medical School, Boston, MA 02115, USA; 5 Department of Pediatrics, Hasbro Children’s Hospital, Warren Alpert Medical School of Brown University, Providence, RI 02912, USA; 6 Neurology 4-Neuroimmunology and Neuromuscular Diseases, Fondazione IRCCS Istituto Neurologico Carlo Besta, 20133 Milan, Italy; 7 Institute of Experimental Genetics, German Mouse Clinic, Helmholtz Center Munich (GmbH), German Research Center for Environmental Health, 85764 Neuherberg, Germany; 8 Institute of Molecular Animal Breeding and Biotechnology, Gene Center, Ludwig Maximilian University of Munich, 81377 Munich, Germany; 9 German Center for Diabetes Research (DZD), 85764 Neuherberg, Germany; 10 Experimental Genetics, TUM School of Life Sciences, Technical University of Munich, 85354 Freising, Germany

**Keywords:** baricitinib, Hutchinson–Gilford progeria syndrome, lamin A, progerin, JAK-STAT, lifespan, inflammation

## Abstract

Hutchinson–Gilford progeria syndrome (HGPS) is a rare, fatal, and premature aging disorder caused by progerin, a truncated form of lamin A that disrupts nuclear architecture, induces systemic inflammation, and accelerates senescence. While the farnesyltransferase inhibitor lonafarnib extends the lifespan by limiting progerin farnesylation, it does not address the chronic inflammation or the senescence-associated secretory phenotype (SASP), which worsens disease progression. In this study, we investigated the combined effects of baricitinib (BAR), a JAK1/2 inhibitor, and lonafarnib (FTI) in a Lmna^G609G/G609G^ mouse model of HGPS. BAR + FTI therapy synergistically extended the lifespan by 25%, surpassing the effects of either monotherapy. Treated mice showed improved health, as evidenced by reduced kyphosis, better fur quality, decreased incidence of cataracts, and less severe dysgnathia. Histological analyses indicated reduced fibrosis in the dermal, hepatic, and muscular tissues, restored cellularity and thickness in the aortic media, and improved muscle fiber integrity. Mechanistically, BAR decreased the SASP and inflammatory markers (e.g., IL-6 and PAI-1), complementing the progerin-targeting effects of FTI. This preclinical study demonstrates the synergistic potential of BAR + FTI therapy in addressing HGPS systemic and tissue-specific pathologies, offering a promising strategy for enhancing both lifespan and health.

## 1. Introduction

The rare genetic disorder Hutchinson–Gilford progeria syndrome (HGPS; OMIM: #176670) is a devastating multi-system disease characterized by accelerated aging in children, leading to premature death in the early teens due to cardiovascular complications, such as myocardial infarction or stroke [1]. In addition to cardiovascular issues, HGPS patients present with symptoms like alopecia, atherosclerosis, lipodystrophy, dysgnathia, and arthritis, many of which overlap with normal aging [2]. Most children with HGPS die before reaching reproductive age [3,4].

A variety of genetic origins of HGPS have been described, but the most common cause of HGPS is a de novo heterogeneous G608G point mutation in the *LMNA* gene (c.1824C>T; GGC>GCT) [5,6,7]. This mutation leads to the production of a truncated prelamin A protein, known as progerin. Progerin integrates into the nuclear lamina, leading to the disruption of the nuclear envelope and causing an abnormal nuclear shape and gene expression, mitochondrial dysfunction, premature senescence, and increased DNA damage [2,8,9,10,11,12].

In healthy cells, the *LMNA* gene encodes the lamin isoforms prelamin A and lamin C [13]. While lamin C is considered mature after translation, prelamin A undergoes a series of post-translational modifications, including farnesylation, proteolytic cleavage of the farnesyl group by RCE1 or ZMPSTE24, carboxymethylation, and a final proteolytic removal of the last 15 amino acids [14,15,16]. Progerin lacks a second endoproteolytic cleavage site and remains permanently farnesylated, resulting in its persistent attachment to the nuclear envelope [17,18,19,20].

Despite its rarity, HGPS provides valuable insights into aging mechanisms and highlights the urgent need for effective treatments targeting both nuclear defects and the associated inflammatory pathways. To date, only one pharmaceutical treatment for HGPS has been approved by the Food and Drug Administration [21]. The farnesyltransferase inhibitor lonafarnib has demonstrated efficacy in increasing the lifespan of patients with HGPS by up to 20% [21,22]. Lonafarnib reduces the farnesylation of prelamin A by anchoring to the nuclear envelope and delaying disease progression. However, lonafarnib is not a complete cure and has significant side effects at both the systemic and cellular levels [23,24,25,26]. It has been shown to trigger genomic instability and increase innate immune responses to self-DNA in the cytoplasm [27,28]. Specifically, cyclic-GAMP synthase (cGAS) generates cGAMP upon DNA binding, thereby activating the adaptor protein STING [29]. STING promotes the nuclear translocation of IRF3 and NF-κB, inducing type I interferons (IFNs) and other proinflammatory cytokines [30,31]. These cytokines activate the JAK/STAT pathway, which not only drives the expression of genes involved in pathogen clearance but also contributes to senescence induction [32,33,34,35,36].

Although lonafarnib offers therapeutic benefits, it also has significant negative side effects, making it a double-edged sword for the treatment of HGPS [27]. On the one hand, it ameliorates HGPS symptoms by decreasing progerin levels and progerin’s incorporation at the nuclear envelope [37]. On the other hand, prenylation is a critical cellular process that affects various substrates, including lamins, Ras, Rheb, and many others [38,39]. Farnesyltransferase inhibition disrupts these processes, leading to cellular side effects such as binucleated cells and donut-shaped nuclei [25,26]. Moreover, lonafarnib-induced genomic instability exacerbates the innate immune response, further accelerating aging [40].

The targeted inhibition of the JAK/STAT pathway is a potential strategy for reducing cytokine-induced stress and counteracting lonafarnib-related side effects, thereby providing a more balanced therapeutic approach. The upregulation of proinflammatory cytokines in human HGPS fibroblasts, along with the chronic inflammation observed in age-associated diseases, such as vascular disease and arthritis, supports the hypothesis that the JAK/STAT pathway plays a role in HGPS progression [41,42]. The JAK/STAT signaling pathway regulates key processes, such as cell death, tumor formation, cellular immunity, and inflammation [43,44,45].

Considering the important role of inflammation in HGPS pathology, strategies targeting both progerin accumulation and inflammation have gained increasing attention. Recent studies have demonstrated that baricitinib, a JAK1/2 inhibitor approved by the FDA for the treatment of rheumatoid arthritis [46], can ameliorate the HGPS cell phenotype [47]. Baricitinib reduces the JAK/STAT pathway overactivation and mitigates the senescence-associated secretory phenotype (SASP) in HGPS fibroblasts [47]. Moreover, our findings showed that a combination of lonafarnib and baricitinib effectively restores cellular homeostasis and reduces inflammation [27]. Notably, baricitinib also alleviates the negative side effects of lonafarnib treatment, including misshapen nuclei and the overactivation of the cGAS-STING-STAT1 signaling axis [27].

Building on these findings, we investigated the therapeutic potential of this combination approach in a preclinical context. In vitro results motivated the transition to a murine model of HGPS (Lmna^G609G/G609G^) [48], which was used to test the drug combination of lonafarnib and baricitinib. The Lmna^G609G/G609G^ mouse model closely mimics human HGPS and exhibits numerous phenotypic similarities, including reduced levels of intact lamin A, progerin accumulation, nuclear alterations, and key clinical features, such as decreased lifespan, altered metabolism, cardiovascular and osteogenic pathologies, and reduced body size [48].

Organs differ in their susceptibility to the pathological effects of progerin expression [49]. Lamin A expression has been shown to increase with tissue stiffness [41,50]. In patients with HGPS, the most pronounced deterioration has been observed in stiffer tissues, such as bone, skin, and muscle, as well as in the vascular system, which comprises endothelial cells (ECs) and vascular smooth muscle cells (VSMCs) [51].

This study demonstrated, for the first time, that combining baricitinib and lonafarnib (referred to as BAR + FTI) produces synergistic benefits that surpass those observed with either baricitinib (BAR) or lonafarnib (FTI) monotherapy. Specifically, BAR + FTI treatment significantly improved survival time, macrophysiological parameters, systemic inflammation, and cellular senescence markers in the treated cohort.

## 2. Results

### 2.1. Baricitinib and Lonafarnib Combination Therapy Extends Survival and Improves Systemic Health

Progeroid mice of the Lmna^G609G/G609G^ genotype [48] were subjected to a comparative evaluation of therapeutic effects across different treatment groups (MOCK WT = untreated Lmna^+/+^ mice, MOCK HOM = untreated Lmna^G609G/G609G^ mice, BAR = baricitinib-treated Lmna^G609G/G609G^ mice, FTI = lonafarnib-treated Lmna^G609G/G609G^ mice, BAR + FTI = baricitinib- and lonafarnib-treated Lmna^G609G/G609G^ mice). This study assessed key parameters critical to progeria pathology, including survival time, systemic health, glucose metabolism, and organ morphology, to validate the therapeutic efficacy of single and combination treatments.

**Figure 1:**
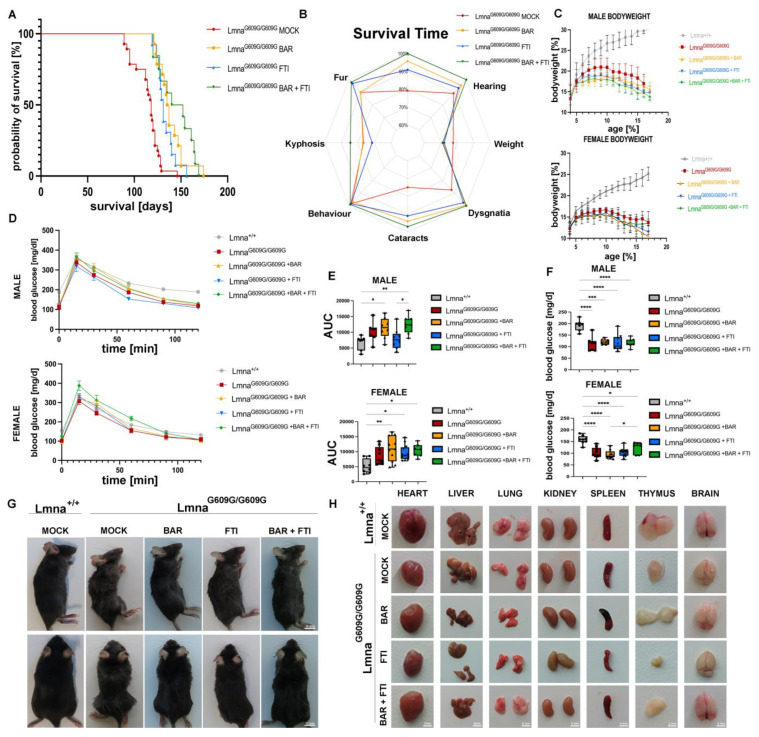
BAR + FTI therapy extends survival, enhances systemic health, and partially restores organ morphology in Lmna^G609G/G609G^ mice. (**A**): Kaplan–Meier graph showing the survival of Lmna^G609G/G609G^ mice across different treatment cohorts over time (MOCK: *n* = 28; BAR: *n* = 14; FTI: *n* = 13; BAR + FTI: *n* = 12). The MOCK HOM cohort showed the shortest lifespan, whereas the BAR + FTI combination displayed the most significant improvement in survival, surpassing both single treatments (BAR or FTI). (**B**): Radar chart illustrating systemic health parameters across different treatment cohorts. Each axis represents the degree of the effect observed for each health parameter. This chart allows for a direct visual comparison of health parameters. Larger enclosed areas indicate better systemic health. MOCK HOM mice showed the most severe deterioration in health parameters. The BAR + FTI-treated mice exhibited the most substantial overall improvements (MOCK: *n* = 28; BAR: *n* = 12; FTI: *n* = 13; BAR + FTI: *n* = 12). (**C**): Body weight changes over time in male (**top plot**) and female (**bottom plot**) mice. Color code is as shown in (**A**). All progeroid groups showed a progressive decline in body weight starting at 8–10 weeks of age (male: MOCK WT: *n* = 13; MOCK HOM: *n* = 11; BAR: *n* = 7; FTI: *n* = 6; BAR + FTI: *n* = 8; female: MOCK WT: *n* = 12; MOCK HOM: *n* = 18; BAR: *n* = 7; FTI: *n* = 7; BAR + FTI: *n* = 6). (**D**): Glucose tolerance was assessed using intraperitoneal glucose tolerance tests (ipGTTs) in male (**top**) and female (**bottom**) mice. MOCK HOM mice exhibited impaired glucose clearance. Glucose intolerance was further exacerbated in the BAR and BAR + FTI groups compared to that in the FTI alone group (males: MOCK WT: *n* = 8; MOCK HOM: *n* = 7; BAR: *n* = 5; FTI: *n* = 9; BAR + FTI: *n* = 8; females: MOCK WT: *n* = 9; MOCK HOM: *n* = 9; BAR: *n* = 6; FTI: *n* = 6; BAR + FTI: *n* = 7). (**E**): Quantification of glucose tolerance (ipGTT) as area under the curve (AUC) for male (**top**) and female (**bottom**) cohorts. Glucose tolerance was further reduced in the BAR and BAR + FTI cohorts but improved in the FTI single treatment (male: MOCK WT: *n* = 8; MOCK HOM: *n* = 7; BAR: *n* = 5; FTI: *n* = 9; BAR + FTI: *n* = 8; female: MOCK WT: *n* = 9; MOCK HOM: *n* = 9; BAR: *n* = 6; FTI: *n* = 6; BAR + FTI: *n* = 7). (**F**): Fasting blood glucose levels in male (**top**) and female (**bottom**) mice after a 6 h fasting period. All treatment groups with the Lmna^G609G/G609G^ genotype showed reduced fasting glucose levels compared with Lmna^+/+^ mice (male: MOCK WT: *n* = 8; MOCK HOM: *n* = 7; BAR: *n* = 5; FTI: *n* = 9; BAR + FTI: *n* = 8; female: MOCK WT: *n* = 9; MOCK HOM: *n* = 9; BAR: *n* = 6; FTI: *n* = 6; BAR + FTI: *n* = 7). (**G**): Representative photographs at 90 days of age showing systemic health differences between the progeroid-treated groups. MOCK HOM mice showed severe kyphosis, fur abnormalities, and reduced body size. BAR and FTI treatments partially alleviated these symptoms, whereas the BAR + FTI treatment resulted in the most pronounced improvements. (**H**): Representative images of dissected organs at 90 days of age across the different cohorts. MOCK HOM mice exhibited a marked reduction in organ size, particularly in the spleen and thymus. FTI treatment further reduced the size of these organs, whereas BAR + FTI treatment improved their size and morphology. Detailed organ weight data are shown in Supplemental Figure S1. (* *p* < 0.05; ** *p* < 0.01; *** *p* < 0.001; **** *p* < 0.0001; ordinary one-way ANOVA test with Tukey’s post hoc test).

A Kaplan–Meier survival analysis (Figure 1A) showed significant differences in survival time among the treatment cohorts. The MOCK HOM mice had an average survival time of 114,36 days (*n* = 28). Both single treatments significantly extended survival time (a significant increase in survival time was considered an increase of at least 10%). The BAR group showed an average survival time of 138.36 days (*n* = 14), representing a 21% increase, and the FTI cohort reached 131.31 days (*n* = 13), indicating a 14.82% increase. Importantly, the BAR + FTI combination therapy achieved the greatest increase in survival, with an average of 142.43 days (*n* = 14), corresponding to a 24.55% increase, surpassing both the single treatments and the synergistic potential of the combination therapy (Figure 1A).

Throughout their lifetimes, we monitored a set of health parameters in all the treatment cohorts (Figure 1B). These health metrics were summarized in a radar chart to illustrate the overall health benefits of each treatment (Figure 1B). The MOCK HOM mice displayed the most severe deterioration in health, as indicated by the smallest enclosed area on the chart. The key deficits included reduced survival time, pronounced cataracts, and dysgnathia. Treatment with BAR or FTI partially improved these parameters. Importantly, the BAR + FTI combination restored systemic health to the greatest extent, as indicated by the largest enclosed area on the radar chart, underscoring its superior efficacy (Figure 1B). Body weight measurements over time (Figure 1C) showed no improvement in relative weight gain in any treatment group compared to the HOM MOCK group. All the treated cohorts showed progressive weight loss, suggesting that none of the therapies directly influenced this parameter.

Glucose metabolism was evaluated using an intraperitoneal glucose tolerance test (ipGTT; Figure 1D). MOCK HOM mice showed impaired glucose clearance compared with wild-type controls, reflecting substantial metabolic dysfunction. Compared with the FTI monotherapy, the BAR and BAR + FTI cohorts showed further reductions in glucose tolerance. Quantification of the ipGTT results using area under the curve (AUC) analysis (Figure 1E) confirmed that the BAR and BAR + FTI treatments resulted in the most pronounced impairments in glucose metabolism, whereas FTI treatment alone improved the AUC values for both male and female mice (Figure 1E).

Fasting blood glucose levels (Figure 1F) were reduced in all treatment groups compared to the wild-type controls, with no significant differences observed between the HOM MOCK and treatment cohorts. These findings suggest that fasting glucose levels are minimally affected by these therapies. Further analysis of blood plasma from MOCK HOM mice showed significant reductions in insulin, FGF-21, non-fasting glucose, triglyceride, total cholesterol, and HDL levels and increased LDL and beta-hydroxybutyrate levels (Figure S1). These metabolic alterations were more pronounced in male mice, highlighting potential sex-dependent differences in metabolic disruption. Treatment groups, including BAR, FTI, and BAR + FTI, did not exhibit statistically significant improvements in these parameters compared to HOM mock mice. However, moderate improvements in insulin and non-fasting glucose levels were observed in the BAR + FTI male cohort, suggesting a potential synergistic effect of the combination therapy in mitigating metabolic dysfunction (Figure S1).

Representative images at 90 days of age (Figure 1G) show severe kyphosis, fur abnormalities, and reduced body size in MOCK HOM mice. The BAR and FTI treatments partially improved these phenotypes, whereas the BAR + FTI combination resulted in the most pronounced improvement, nearly normalizing the outward appearance at this time point (Figure 1G). An organ morphology assessment indicated substantial reductions in the sizes of the spleen and thymus in MOCK HOM mice (Figure 1H). Treatment with FTI alone further aggravated the reduction in organ size, highlighting its potential deleterious effects on certain tissues. In contrast, the BAR and BAR + FTI treatments partially restored organ morphology and size. These results highlight the ability of BAR + FTI therapy to mitigate progeroid organ deterioration. Brain morphology remained unaffected in all the treatment groups. The detailed organ weight data are presented in Figure S2.

### 2.2. The Baricitinib and Lonafarnib Combination Reduces STAT1/STAT3 Activation and Progerin Accumulation in Lmna^G609G/G609G^ Mice

We previously reported that STAT1 and STAT3 were activated in fibroblasts from HGPS patients [27]. To evaluate the status of STAT1 and STAT3 activation in the Lmna^G609G/G609G^ mouse model, we performed western blot analyses on multiple tissues commonly affected by progeria.

**Figure 2:**
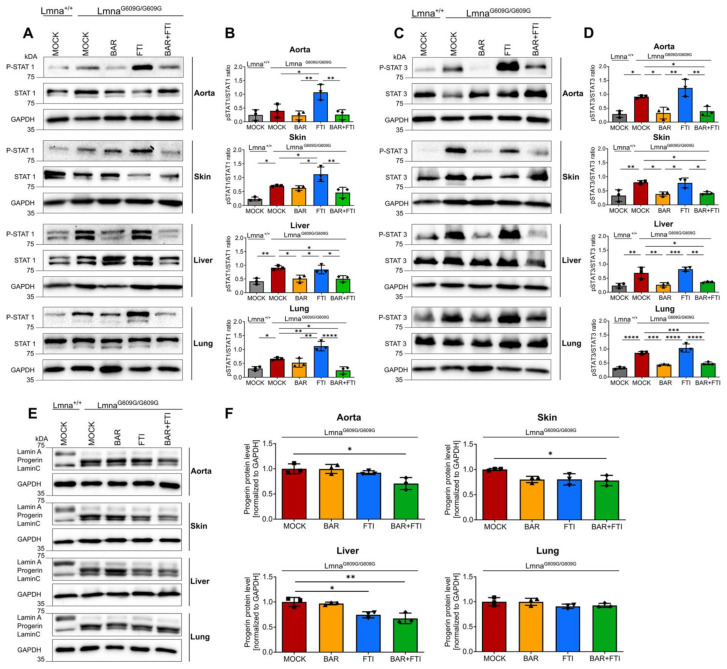
Combination therapy reduces STAT1/STAT3 activation and progerin accumulation in Lmna^G609G/G609G^ mice. (**A**,**C**,**E**): Representative images of western blots for P-STAT1, STAT1, P-STAT3, STAT3, lamin A/C, and progerin extracted from the aorta, skin, liver, and lung tissues of 90-day-old Lmna^+/+^ and Lmna^G609G/G609G^ mice (**B**,**D**,**F**): Quantification of P-STAT1/STAT1 ratios (**B**), P-STAT3/STAT3 ratios (**D**), and progerin levels (**F**). Graphs show mean ± SD. (*n* = 3); * *p* < 0.05, ** *p* < 0.01, *** *p* < 0.001, **** *p* < 0.0001; ordinary one-way ANOVA test with Tukey’s post hoc test.

On day 90, Lmna^G609G/G609G^ mice showed substantially greater STAT1 activation (P-STAT1/STAT1 ratio) in the skin, liver, and lungs than wild-type mice, with the aorta showing a non-significant trend toward increased activation (Figure 2A,B). Treatment with FTI alone further increased STAT1 activation in the aorta, skin, and lungs compared to mock-treated Lmna^G609G/G609G^ mice (Figure 2A,B). This observation highlights the role of FTI in amplifying harmful inflammatory pathways. In contrast, BAR treatment robustly suppressed FTI-induced activation of STAT1 across all tested organs, restoring the activation levels to near-normal levels in several tissues.

Similarly, STAT3 activation (P-STAT3/STAT3 ratios) was significantly elevated in all examined organs of Lmna^G609G/G609G^ mice compared to wild-type mice (Figure 2C,D). BAR treatment, either as a monotherapy or in combination with FTI, effectively normalized P-STAT3/STAT3 ratios to levels comparable to those in wild-type mice. In contrast, FTI alone did not reduce STAT3 activation. Instead, FTI treatment even slightly increased the P-STAT3/STAT3 ratio in the aorta, liver, and lungs compared to that in mock Lmna^G609G/G609G^ mice (Figure 2C,D). These findings underscore BAR’s ability to counteract FTI-induced STAT1 hyperactivation and its unique efficacy in normalizing aberrant STAT3 signaling, particularly in tissues such as the aorta and skin, which are the most affected in HGPS.

To further assess the efficacy of the combination treatment, we measured progerin protein levels (Figure 2E). As expected, the progerin signal was undetectable in Lmna^+/+^ mice (Figure 2E). In Lmna^G609G/G609G^ mice, both BAR and FTI monotherapy reduced progerin levels in the aorta, skin, and liver (Figure 2E,F). Importantly, the combination of BAR and FTI resulted in the greatest increase in progerin clearance beyond that was achieved with either treatment alone (Figure 2F).

### 2.3. Baricitinib and Lonafarnib Therapy Reduces Tissue Degeneration and Fibrosis in Lmna^G609G/G609G^ Mice

**Figure 3:**
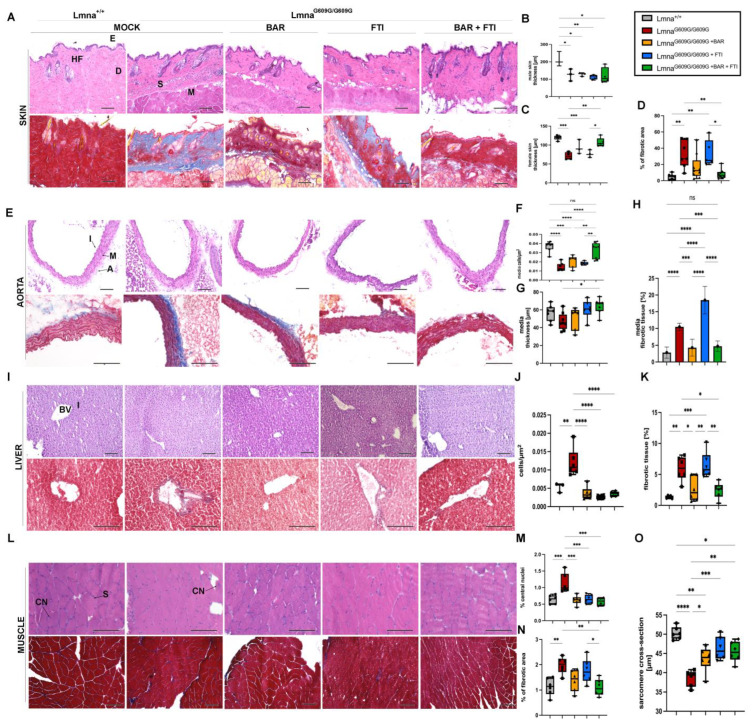
BAR + FTI combination improves progerin-induced tissue degeneration and fibrosis. (**A**,**E**,**I**,**L**): Histological analysis of skin (**A**), aorta (**E**), liver (**I**), and muscle (**L**) using hematoxylin and eosin (**H**,**E**, upper row) and Masson’s trichome staining (lower row), scale bar = 100 μm. In panel (**A**), HF denotes hair follicle, E epidermis, D dermis, S subdermis, M muscular layer. In panel (**E**), I denotes intima, M media, A adventitia. In panel (**I**), BV = blood vessel, I = interstitium and in (**L**) CN = central nucleus, S = sarcomere. MOCK-treated Lmna^G609G/G609G^ mice exhibited severe tissue degeneration and fibrosis across all tissues. (**B**–**D**): Quantification of skin parameters. Skin thickness in male (**B**) and female (**C**) mice is significantly reduced in MOCK HOM group, which was partially restored in BAR and BAR + FTI cohorts (male: MOCK WT: *n* = 3; MOCK HOM: *n* = 3; BAR: *n* = 3; FTI: *n* = 5; BAR + FTI: *n* = 4; female: MOCK WT: *n* = 5; MOCK HOM: *n* = 4; BAR: *n* = 3; FTI: *n* = 3; BAR + FTI: *n* = 5). (**D**): Dermal fibrosis is elevated in MOCK HOM mice and reduced in BAR + FTI cohorts compared to those receiving single treatments (MOCK WT: *n* = 7; MOCK HOM: *n* = 7; BAR: *n* = 9; FTI: *n* = 5; BAR + FTI: *n* = 8). (**F**–**H**): Quantification of aorta parameters. Media cellularity that is reduced in MOCK HOM mice is restored in BAR + FTI-treated animals (MOCK WT: *n* = 7; MOCK HOM: *n* = 7; BAR: *n* = 6; FTI: *n* = 6; BAR + FTI: *n* = 8). (**G**): Media thickness reduced in MOCK HOM animals is partially restored in BAR + FTI-treated mice (MOCK WT: *n* = 7; MOCK HOM: *n* = 8; BAR: *n* = 6; FTI: *n* = 6; BAR + FTI: *n* = 7). (**H**): Aortic media fibrosis, which is elevated in MOCK HOM- and FTI-treated mice, is reduced in BAR and FTI + FTI cohorts, with no marked improvement in FTI cohorts (MOCK WT: *n* = 6; MOCK HOM: *n* = 8; BAR: *n* = 6; FTI: *n* = 6; BAR + FTI: *n* = 6). (**J**,**K**): Hepatic tissue analysis. Increased cellularity in the MOCK HOM cohort, which is indicative of reduced hepatocyte size, is partially reduced in all treatment groups (MOCK WT: *n* = 3; MOCK HOM: *n* = 6; BAR: *n* = 6; FTI: *n* = 6; BAR + FTI: *n* = 6). (**K**): Fibrosis in hepatic tissue, which is most pronounced in the MOCK HOM- and FTI-treated groups, is significantly reduced in the BAR and BAR + FTI cohorts, with BAR + FTI showing the most efficacy (MOCK WT: *n* = 5; MOCK HOM: *n* = 6; BAR: *n* = 6; FTI: *n* = 6; BAR + FTI: *n* = 6). (**M**–**O**): Muscle tissue changes: Central nucleation is elevated in MOCK HOM mice and reduced in all treatment groups. Muscle fibrosis is pronounced in HOM MOCK and FTI mice but reduced in BAR + FTI mice. The sarcomere diameter is reduced in MOCK HOM mice and fully restored with FTI and BAR + FTI treatments (MOCK WT: *n* = 6; MOCK HOM: *n* = 6; BAR: *n* = 6; FTI: *n* = 6; BAR + FTI: *n* = 6) (* *p* < 0.05; ** *p* < 0.01; *** *p* < 0.001; **** *p* < 0.0001; ordinary one-way ANOVA test with Tukey’s post hoc test).

A histological examination of the skin, aorta, liver and muscle tissues revealed extensive tissue degeneration and fibrosis in MOCK Lmna^G609G/G609G^ mice at 90 days of age (Figure 3). These phenotypic changes were mitigated to a certain degree by BAR, FTI, and their combination. MOCK HOM mice exhibited reduced skin thickness compared with control WT-type mice (Figure 3B,C). This deficit was partially restored in the BAR + FTI cohort, underscoring the enhanced efficacy of the combination treatment for skin tissue repair (Figure 3B,C). Elevated dermal fibrosis detected in the MOCK HOM group was significantly reduced by BAR treatment and was nearly completely reversed by BAR + FTI treatment (Figure 3D). In contrast, FTI monotherapy failed to improve dermal fibrosis, indicating its limited efficacy in restoring this parameter (Figure 3D).

In the aorta, media cellularity and thickness were significantly reduced in MOCK HOM mice, indicating severe vascular atrophy, as reported previously [52,53] (Figure 3E,H). This reduction reflects the profound structural and functional changes associated with progerin expression. Although all treatment groups showed some improvement, BAR + FTI therapy resulted in the most pronounced restoration of both media thickness and cellularity (Figure 3F,G). Media fibrosis was strongly increased in both MOCK HOM- and FTI-treated mice but was significantly reduced with BAR and BAR + FTI treatments (Figure 3H). BAR + FTI therapy achieved the most substantial reduction in vascular fibrosis, emphasizing its therapeutic potential for vascular degeneration.

Additionally, electrocardiography (ECG) and transthoracic echocardiography (TTE) were performed as single measurements in awake mice at 90 days of age across all cohorts to assess the potential cardiac effects of the treatments. While ECG and TTE suggested the beneficial effects of the therapies, high intergroup variability limited the ability to detect statistically significant differences (Figures S3 and S4). Moreover, substantial differences in body weight across groups necessitated its inclusion as a covariate in the analysis. The limitations of our experimental approach are discussed in the Section 3.

Liver tissues from the MOCK HOM cohort showed increased cellularity, indicating reduced hepatocyte size (Figure 3I,J). This parameter was significantly improved in all treatment groups, suggesting that both the BAR and FTI therapies promote hepatocyte restoration. However, hepatic vascular fibrosis, which was most pronounced in MOCK-HOM-treated mice, did not improve after FTI therapy (Figure 3K). In contrast, the BAR and BAR + FTI treatments significantly reduced fibrosis levels (Figure 3H).

A muscle tissue analysis showed an increased number of central nuclei in the fibers in the MOCK HOM group, which is a hallmark of muscle degeneration (Figure 3L,M) [54,55]. All the treatment groups showed a reduction in this parameter, indicating the treatments’ capacity to mitigate muscle degeneration. Increased fibrosis in the muscle tissue was also observed in the MOCK HOM cohort (Figure 3N). Although FTI treatment did not improve muscle fibrosis, the BAR and BAR + FTI cohorts showed significant reductions in fibrosis levels, which were restored to near-wild-type levels (Figure 3N). the sarcomere diameter was reduced in MOCK HOM mice (Figure 3O). The BAR treatment partially restored this parameter, whereas the FTI and BAR + FTI treatments fully restored the sarcomere diameter to levels comparable to those of the wild-type controls (Figure 3O).

Collectively, these histological findings demonstrate that BAR + FTI combination therapy provides the most significant benefits across multiple tissues, including the skin, aorta, liver, and muscle, in Lmna^G609G/G609G^ mice. Although FTI monotherapy failed to reduce fibrosis and even increased it in some tissues, the BAR treatment effectively reduced fibrosis levels in the tissues analyzed. Moreover, the FTI treatment alone showed unique benefits in improving the sarcomere diameter (Figure 3O) and aorta media thickness (Figure 3G), parameters for which BAR alone showed limited efficacy. These results highlight the benefits of a combined treatment approach to address the wide spectrum of tissue damage induced by progerin expression.

### 2.4. BAR + FTI Therapy Mitigates Proinflammatory Profiles, SASP, and ECM Remodeling Across Organ Systems

To further validate the synergistic effects of BAR and FTI combination therapy, we examined the expression profiles of inflammatory, senescence-associated secretory phenotype (SASP), angiogenesis, and extracellular matrix (ECM)-related markers. Our analysis revealed distinct organ- and treatment-specific variations across the observed groups.

**Figure 4:**
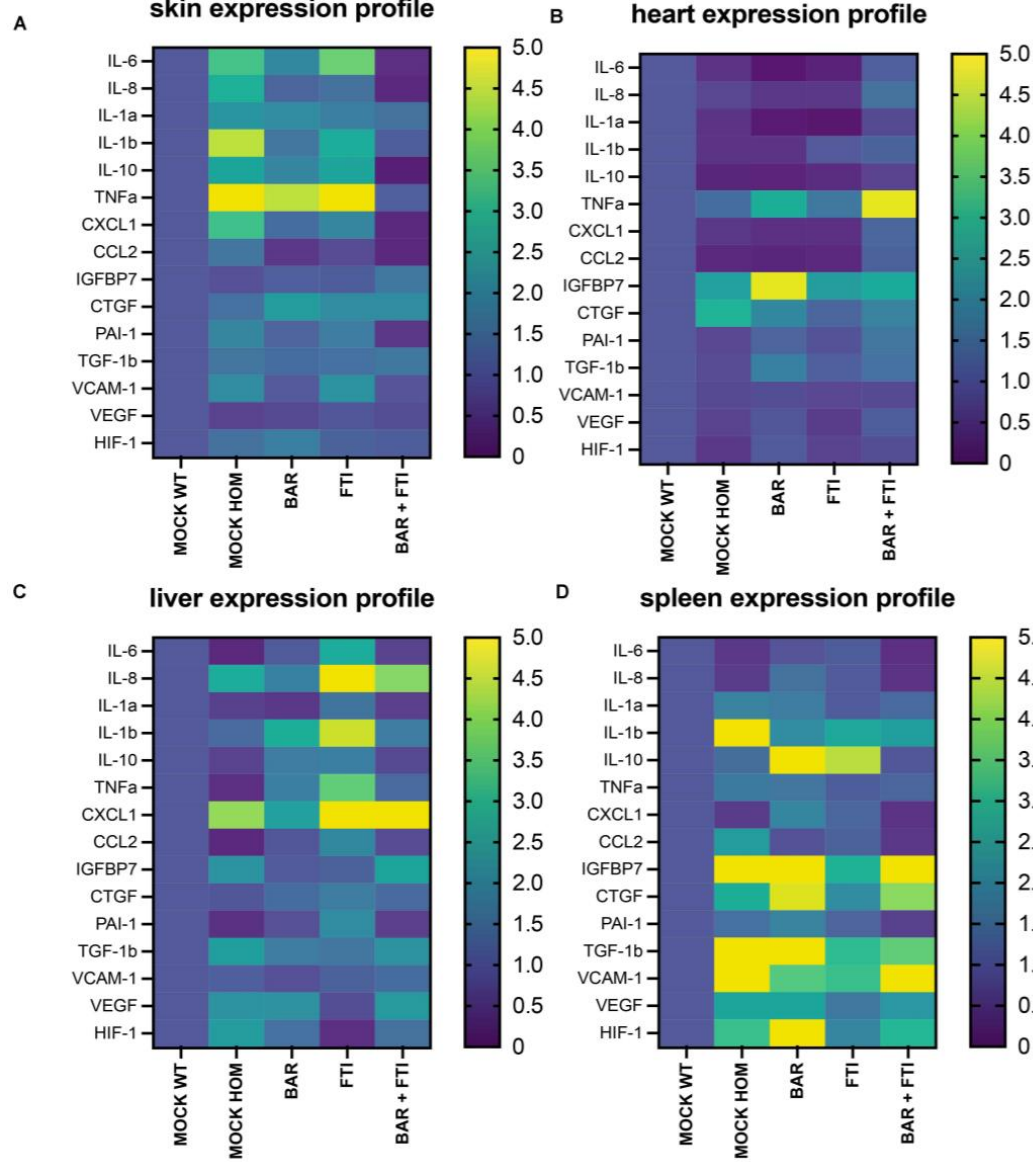
Heatmaps displaying differential mRNA expression across five treatment groups. Each profile represents the relative expression levels of the individual mRNA. Data are presented on a color scale ranging from 0 (dark purple = low expression) to 5 (yellow = high expression). The key markers analyzed include senescence-associated secretory phenotype (SASP), cytokines, and angiogenic and extracellular matrix (ECM)-related markers. The four organs examined are the (**A**) skin, (**B**) heart, (**C**) liver, and (**D**) spleen. The mRNA levels analyzed include IL-6, IL-8, IL-1a, IL-1b, IL-10, TNFα, PAI-1, TGF-1b, CXCL1, CCL2, IGFBP7, VCAM-1, CTGF, VEGF, and HIF-1. Fold changes are shown relative to the MOCK WT cohort. Data represent a minimum of *n* = 3 biological replicates per organ and treatment group.

In the skin tissue (Figure 4A), the MOCK HOM cohort exhibited marked increases in the levels of inflammatory cytokines (IL-6, IL-8, IL-1β, and TNFα) and SASP markers, such as PAI-1, CXCL1, and CCL2, indicating a proinflammatory and senescent environment (Figure 4A).

BAR and FTI monotherapy partially reduced the levels of these inflammatory markers, with BAR showing the greatest efficacy. Notably, BAR + FTI therapy resulted in the most significant reduction in inflammatory and SASP marker levels, effectively normalizing these profiles (Figure 4A). Markers of extracellular remodeling, including VCAM-1 and CTGF, were reduced by the BAR + FTI treatment, whereas FTI alone had no effect (Figure 4A). The substantial reduction in IGFBP7, VCAM-1, and CTGF expression further highlights the potential of BAR + FTI in mitigating inflammation, senescence, and ECM remodeling in the skin.

In the cardiac tissue (Figure 4B), the MOCK HOM cohort showed decreased levels of inflammatory cytokines (IL-6, IL-8, IL-1β, and TNFα) and angiogenesis markers (VEGF and HIF-1. The BAR and FTI treatments modestly increased the expression of these markers, with FTI resulting in greater normalization of inflammatory and angiogenic profiles. The most pronounced restoration of these markers was observed in the BAR + FTI cohort, indicating their synergistic potential (Figure 4B). TNFα expression was elevated in both the BAR and BAR + FTI cohorts, whereas VCAM-1 levels remained mostly unchanged across groups. The upregulation of CTGF and IGFBP7 in MOCK HOM mice was significantly reduced by BAR + FTI treatment (Figure 4B). These findings demonstrate that BAR + FTI therapy effectively improves both the inflammatory and ECM remodeling profiles in the hearts of progeroid mice.

In the liver tissue (Figure 4C), MOCK HOM mice exhibited elevated expressions of inflammatory markers (IL-8 and IL-1b) and SASPs (CXCL1), whereas the expression of IL-6 and TNFα was reduced. Angiogenesis markers are upregulated in MOCK HOM mice. The BAR treatment moderately reduced inflammation, SASPs, and angiogenesis markers, whereas the FTI treatment significantly increased these markers, which is consistent with FTI-induced hepatic inflammation [56] (Figure 4C). Importantly, BAR + FTI therapy almost completely normalized the SASP, angiogenesis, ECM, and inflammatory marker levels, demonstrating its therapeutic efficacy in reducing both disease-related and FTI-induced hepatic inflammation.

In the splenic tissue (Figure 4D), MOCK HOM mice exhibited increased levels of inflammatory cytokines and SASP markers, which were marginally reduced by single treatments. The BAR + FTI cohort showed the most significant reduction in these markers. ECM and angiogenesis markers, which were strongly elevated in HOM MOCK mice, were most effectively reduced by the FTI treatment (Figure 4D). However, the BAR + FTI treatment resulted in broader and more balanced reductions in the levels of all inflammatory markers, SASP, ECM, and angiogenesis markers, indicating its superior therapeutic effect.

Additionally, blood cytokine analysis (Figure S5) revealed a generalized increase in cytokine levels in the MOCK HOM cohort. While all treatment groups reduced cytokine levels to some degree, the FTI treatment markedly elevated the IL-1β and IL-6 levels compared to the MOCK HOM group, which is consistent with its inflammatory side effects. In contrast, the BAR + FTI treatment resulted in the most consistent and substantial reduction in blood cytokine levels across all markers. Immunoglobulin analysis of the blood plasma showed no significant differences between the groups (Figure S6).

Collectively, these findings demonstrate the consistent efficacy of the BAR + FTI treatment in reducing inflammation, SASP, and ECM remodeling across multiple tissues, with the most significant improvement in the skin, liver, and heart. Importantly, BAR + FTI therapy mitigated the proinflammatory milieu associated with FTI monotherapy in hepatic tissue, further emphasizing the advantages of combination therapy in addressing both disease-related and treatment-induced pathologies.

### 2.5. Baricitinib and Lonafarnib Therapy Mitigates Senescence and Structural Damage in Aortic and Renal Tissue

**Figure 5:**
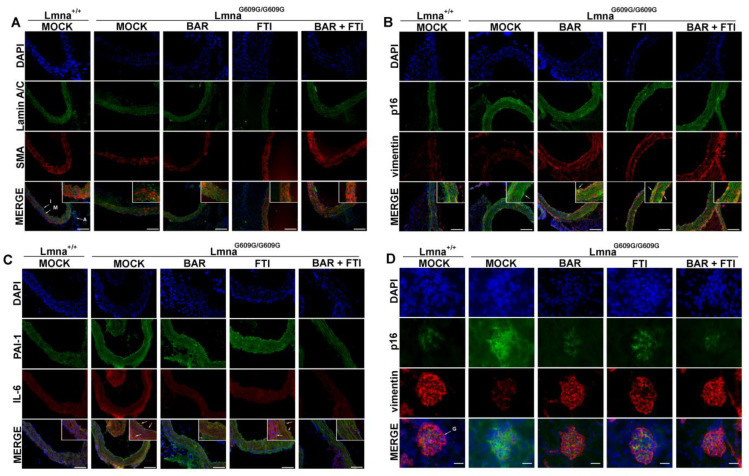
Senescence, ECM remodeling, and inflammation in vascular and renal tissue. (**A**): Immunofluorescent staining in aorta sections at 90 days shows reduced αSMA in MOCK HOM tissue vs. MOCK WT; BAR and FTI monotherapies partially restored αSMA expression, while the BAR + FTI treatment nearly normalized it (*n* = 3). Lamin A/C (green), αSMA (red), and DNA (DAPI, blue) were detected. I denotes intima, M media and A adventitia. (**B**): Staining for the senescence marker p16 (green) and vimentin (red) in aorta sections. There was a high prevalence of p16-positive cells in the intima of HOM MOCK samples (indicated by arrows), which was persistent with FTI, reduced with BAR, and was nearly absent with BAR + FTI. Vimentin was decreased in MOCK HOM and partially restored by treatments (*n* = 3). (**C**): Aortic PAI-1 (green) and IL-6 (red) staining were elevated in MOCK HOM mice, especially in intima and adventitia (indicated by arrows). FTI had little effect, while BAR and BAR + FTI reduced both markers (*n* = 3). (**D**): Immunofluorescent staining in kidney glomeruli (G) showed high expression of senescence marker p16 (green) and reduced vimentin (red) in MOCK HOM mice. BAR and BAR + FTI reduced p16 and increased vimentin; FTI only increased vimentin (*n* = 3). Scale bars: (**A**–**C**): 100 µm; (**D**): 20 µm; blue color represents DAPI counterstaining of nuclei.

An immunofluorescence analysis of aortic and renal tissues was performed to evaluate the markers of inflammation, senescence, and ECM remodeling across the treatment cohorts (Figure 5). In the aorta, MOCK HOM mice showed reduced levels of αSMA, a marker of vascular smooth muscle cells (Figure 5A). Monotherapy with BAR or FTI partially restored αSMA expression, and the BAR + FTI treatment induced the most substantial improvement.

In the aortic intima, MOCK HOM mice showed increased levels of the senescence marker p16 and reduced levels of the mesenchymal marker vimentin, particularly in the media (Figure 5B). Although p16-positive foci persisted in the FTI-treated HOM mice, they were reduced in the BAR-treated samples and were nearly eliminated in the BAR + FTI group. Vimentin expression was reduced in the MOCK-HOM group and partially restored by all three treatments, with BAR + FTI resulting in the most pronounced recovery (Figure 5B).

Markers of inflammation and fibrotic remodeling, including PAI-1 and IL-6, were increased in MOCK HOM mice (Figure 5C). PAI-1 expression was predominantly localized to the aortic intima and adventitia, whereas IL-6 expression was concentrated in the aortic media and adventitia (Figure 5C). BAR monotherapy effectively reduced the expression of both the markers, whereas FTI alone failed to attenuate their elevated expression. The BAR + FTI combination substantially reduced PAI-1 and IL-6 expression across all aortic tissue regions, demonstrating its superior anti-inflammatory and anti-fibrotic potential.

In the renal tissue (Figure 5D), MOCK HOM glomeruli exhibited increased p16 levels and reduced vimentin expression, which is indicative of senescence and structural remodeling. The BAR treatment partially reduced p16 expression and increased vimentin levels, whereas FTI had a minimal effect on p16 but partially restored vimentin expression. Notably, BAR + FTI therapy restored both p16 and vimentin expression to levels comparable to those in wild-type controls.

Overall, these findings demonstrate that the BAR + FTI combination provides the most effective protection against senescence-driven inflammation, fibrosis, and structural remodeling of vascular and renal tissues.

## 3. Discussion

In this preclinical study, we provide robust evidence that the combination of baricitinib, a JAK1/2 inhibitor, and lonafarnib, a farnesyltransferase inhibitor, offers significant therapeutic benefits in delaying the progression of HGPS. Using the Lmna^G609G/G609G^ mouse model [48], we demonstrated that BAR + FTI therapy extended the average lifespan by approximately 25%, surpassing the improvements observed with either monotherapy alone. Additionally, BAR + FTI-treated mice exhibited significant improvements in their health span, including reductions in kyphosis, improved fur quality, reduced cataract incidence, and less severe dysgnathia. Histological analyses showed significant improvements in tissue architecture, including reduced dermal, hepatic, and muscular fibrosis; restored media cellularity and thickness in the aorta; and improved muscular fiber integrity. Furthermore, senescence markers, such as p16 and SASP factors, were significantly decreased, along with reduced inflammatory cytokine expression (e.g., IL-6 and PAI-1) in vascular and renal tissues. These findings underscore the ability of BAR + FTI to address both the systemic and tissue-specific pathologies of HGPS.

The pathogenesis of HGPS is driven by progerin accumulation [49,57,58,59]. Progerin farnesylation causes its anchorage to the nuclear envelope, resulting in alterations in nuclear integrity, the induction of senescence, and the activation of inflammatory pathways [8,27,60,61]. Lonafarnib inhibits farnesyltransferase, reduces progerin farnesylation, and limits its nuclear accumulation [20]. However, this is not sufficient to fully counteract the chronic inflammation and SASPs that drive disease progression.

This study showed that in mock-treated Lmna^G609G/G609G^ mice, STAT1 activation was significantly elevated in the skin, liver, and lungs, with a non-significant trend toward increased activation in the aorta, whereas STAT3 activation was elevated in all examined organs (aorta, liver, lungs, and skin). Notably, FTI treatment further exacerbated STAT1 activation in the aorta, skin, and lungs and failed to reduce STAT3 activation but slightly increased it in the aorta, liver, and lungs. In contrast, BAR effectively suppressed FTI-induced STAT1 hyperactivation in all the tested tissues and normalized STAT3 signaling in the aorta, liver, lungs, and skin. These findings establish, for the first time, that beyond the need to mitigate inflammation driven by progerin accumulation in these affected tissues, it is equally essential to prevent FTI-induced STAT1 and STAT3 hyperactivation. Given that FTI is currently prescribed for HGPS, determining whether baricitinib should be co-administered is a critical clinical question.

Mechanistically, BAR and FTI exerted complementary effects on distinct but interconnected pathogenic pathways. BAR inhibits JAK1/2, thereby effectively disrupting JAK/STAT signaling and reducing the expression of proinflammatory cytokines, SASP factors, and senescence markers, as evidenced in this study and others [27,47,62]. This action mitigates systemic inflammation and cellular senescence, both of which are critical drivers of HGPS pathology [63]. FTI also prevents the farnesylation of progerin and enhances its clearance [37]. BAR + FTI therapy exerts a synergistic effect by simultaneously targeting both the upstream cause (progerin accumulation) and downstream effects (inflammation and senescence). This mechanistic synergy was further observed in the ability of BAR + FTI to not only reduce inflammation and SASP markers aggravated by FTI monotherapy but also mitigate genomic instability, as indicated previously by the reduction in binucleate and donut-shaped nuclei [25,26]. By counteracting FTI’s proinflammatory side effects and enhancing its efficacy in reducing senescence and promoting tissue repair, BAR has emerged as an essential contributor to the overall effectiveness of the combination therapy. These synergistic effects likely explain the superior lifespan and health span improvements observed in BAR + FTI-treated mice.

Lonafarnib (Zokinvy) remains the gold standard for treating HGPS patients due to its ability to extend life expectancy [21]. Emerging therapies, such as gene editing via base editing or antisense oligonucleotides (ASOs), offer promising alternatives [48,64,65]. However, these approaches face challenges, including efficient delivery to targeted tissues, particularly the cardiovascular system, and safety concerns due to off-target effects [66]. Recent advancements include a Phase 2a clinical trial investigating progerinin, a small molecule that inhibits the interaction of progerin with lamin A (https://www.progeriaresearch.org/de/progerinin_trial/, accessed on 12 December 2024). The disruption of this interaction reduces the deleterious effects of progerin and ameliorates nuclear abnormalities and associated dysfunctions [67]. Although progerinin has demonstrated health span benefits in Lmna^G609G/+^ mice, it has not yet been evaluated in combination with lonafarnib. Combining progerinin with lonafarnib is expected to enhance therapeutic efficacy by targeting different stages of the pathogenic pathway of progerin. Given the persistent role of chronic inflammation in HGPS progression, the addition of BAR to this regimen could further improve outcomes by addressing inflammatory pathways that are not targeted by progerinin or FTI. Future studies should investigate the potential of such a triple therapy, combining progerinin, BAR, and FTI, to synergistically reduce progerin accumulation, prevent harmful interactions, and suppress chronic inflammation.

Importantly, BAR, a well-established JAK1/2 inhibitor with a favorable safety profile [68], offers a complementary approach by targeting systemic inflammation, which is a hallmark of HGPS [69,70]. In addition to HGPS, BAR has demonstrated efficacy against a wide spectrum of inflammatory diseases. It is approved by both the FDA and the EMA for the treatment of moderate to severe rheumatoid arthritis in adults, where it alleviates inflammation and mitigates joint damage [71,72,73]. Additionally, BAR is authorized for use in children aged 2 years and older with active juvenile idiopathic arthritis, particularly in cases in which disease-modifying antirheumatic drugs are ineffective or poorly tolerated [73,74]. In the context of juvenile atopic dermatitis, BAR is indicated for children aged 2 years and older who experience inadequate relief from topical therapies, providing effective control of pruritus, erythema, and skin irritation [73,75]. Furthermore, BAR facilitates hair regrowth in alopecia areata, an autoimmune disorder characterized by hair loss [71,73,76]. A unifying feature across these conditions is immune dysregulation, which BAR addresses through the inhibition of the JAK-STAT pathway. By targeting this critical signaling cascade, BAR effectively reduces inflammation and modulates disease progression. Given its established safety and efficacy in pediatric populations and the findings of this study, BAR may be a promising therapeutic option for patients with HGPS. Another advantage of BAR is its oral administration, which offers a convenient once-daily dosing regimen of 2 mg or 4 mg, depending on the patient’s age, weight, and specific disease indications [73].

Despite the promising outcomes, this study has several limitations. Blood cytokine analyses and transthoracic echocardiographic (TTE) measurements were inconclusive, owing to batch effects and the limited sample size, thereby reducing the statistical power to detect subtle but potentially relevant clinical changes. Additionally, the transportation of the mice for cardiac measurements likely induced stress, which further confounded our results. The use of awake electrocardiography (ECG) and TTE introduces a different stress level than measurements performed under sevoflurane anesthesia, as described previously [48]. The vasodilatory effects of sevoflurane and its impact on endothelial function are important factors when comparing results across studies and must be carefully considered in the experimental design and data interpretation. Another inherent challenge in using Lmna^G609G/G609G^ mice is that while they provide a rapid model for screening potential therapies, they also present logistical constraints. Rapid disease progression in these mice facilitates drug testing but limits the ability to assess long-term cardiac function [77]. Moreover, the difficulty of breeding and generating sufficiently homozygous mice in a single cohort further restricts study scalability, as well as the statistical robustness of the study. Additionally, the high cost of lonafarnib, which was generously provided by the Progeria Research Foundation (PRF), constrained both the sample size and experimental scope. Another consideration is the potential for baricitinib-induced immunosuppression via JAK1/2 inhibition. Although no immune-related adverse effects were observed in this study, likely because of the controlled, specific pathogen-free (SPF) environment, future studies should include immune function monitoring to ensure safety in clinical applications.

Overall, this study demonstrates that BAR + FTI therapy effectively targets the key molecular and phenotypic hallmarks of HGPS, significantly enhancing both lifespan and health span. This dual-action therapy offers a synergistic and comprehensive treatment strategy to improve outcomes in patients with HGPS.

## 4. Materials and Methods

### 4.1. Mouse Model and Breeding

Mouse Colony and House Conditions. Progeria phenocopy Lmna^G609G^ mice [48] were kindly provided by Carlos-Lopes Otin (University of Oviedo, Spain). All experiments using this murine model were conducted with the approval of the Bavarian State Government (Regierung von Oberbayern, Munich, Germany; TVA-ID: 55.2-2532.Vet_01-19-72) in accordance with the Animal Welfare Act. Colony initiation was performed as described previously [49]. Sex was used as a biological variable in our study. Sex was also included in the analysis. We observed all parameters in both genders. Eight-week-old female C57BL/6J mice were purchased from Charles River (027C57BL/6; Charles River, Wilmington, MA, USA) and bred with Lmna^G609G/+^ male mice of the same age. To minimize inbreeding, females and males from different breeds were paired. We maintained a colony with at least five distinct family lines to ensure genetic diversity and reduce the risks of inbreeding. Female Lmna^+/+^ offspring were used for the maintenance breeding of Lmna^G609G/+^ males. Heterozygous males and females from different family lines were crossed to produce homozygous (Lmna^G609G/G609G^) offspring for experimental purposes. Offspring resulting from Lmna^G609G/+^ × Lmna^G609G/+^ breeding, regardless of genotype, were not used for colony maintenance. Moreover, Lmna^G609G/+^ breeding animals were not maintained beyond 18 weeks of age. Homozygous Lmna^G609G/G609G^ mice received soaked food starting at 8 weeks of age and were always housed with at least one Lmna^G609G/+^ or Lmna^+/+^ littermate to ensure appropriate care. Both experimental and maintenance animals were housed at controlled temperatures of 21–22 °C under a 12 h light/dark cycle. Standard chow (PS RM-H, V1534; ssniff Spezialdiäten GmbH, Soest, Germany) was provided to all animals. Depending on the treatment group, the standard chow was supplemented with baricitinib (62.5 mg/kg, MedChemExpress, Princeton, NJ, USA; HY-15315), lonafarnib (187.5 mg/kg; kindly provided by the Progeria Research Foundation, Peabody, MA, USA), or a combination of baricitinib (62.5 mg/kg) and lonafarnib 187.5 mg/kg lonafarnib).

### 4.2. Intraperitoneal Glucose Tolerance Test (ipGTT)

The mice were subjected to 6 h of fasting prior to the glucose tolerance test. During fasting, the food was removed, and the mice were placed in clean cages with permanent access to fresh water. To minimize stress, mice were acclimatized to the testing environment for 1 h before the procedure. Each mouse received an intraperitoneal injection of glucose solution (2 g/kg bodyweight; G-20% glucose injection solution; Braun, Melsungen, Germany), and blood samples were collected and measured using a blood glucose meter (Contour Next One; Ascensia, Parsippany, NJ, USA) at 0, 15, 30, 60, 90, and 120 min post-injection. Mice were held in a mouse restrainer with dorsal access (SP Scienceware No.:464010000) for blood sample acquisition. Glucose tolerance was assessed by quantifying the area under the curve (AUC) of the blood glucose concentration over time. Baseline glucose levels were subtracted to generate an area over the curve (AOC), which provided a quantitative measure of glucose tolerance.

### 4.3. Western Blot Analysis

Mouse tissues were immediately snap-frozen in liquid nitrogen and homogenized in RIPA lysis buffer supplemented with PMSF, protease inhibitors, and phosphatase inhibitors using a Dounce homogenizer (Sigma Aldrich, St. Louis, MO, USA). The homogenized samples were centrifuged at 13,000× *g* for 15 min at 4 °C, and the total protein concentrations were measured using the Bradford assay (Bio-Rad Laboratories, Inc., Hercules, CA, USA). Equal amounts of protein were loaded onto SDS-polyacrylamide gels (8% or 10%), followed by wet transfer onto nitrocellulose membranes. Membranes were blocked for 1 h at room temperature with 5% non-fat milk and incubated overnight at 4 °C with the following antibodies: anti-P-STAT1 (9167, Cell Signaling, 1:800) (Danvers, MA, USA), anti-STAT1 (14994, Cell Signaling, 1:1000), anti-P-STAT3 (9145, Cell Signaling, 1:1000), anti-STAT3 (9139, Cell Signaling, 1:1000), anti-lamin A/C (sc20681, Santa Cruz Biotechnology, 1:5000) (Dallas, TX, USA), and anti-GAPDH (G9545, Sigma Aldrich, 1:10,000). Following incubation, the membranes were washed three times with TBS-Tween and incubated with horseradish peroxidase-conjugated secondary antibodies (anti-rabbit or anti-mouse, 1:5000 Jackson ImmunoResearch Laboratories) (Philadelphia, PA, USA). After washing with TBS-Tween, the signals were visualized using enhanced chemiluminescence (ECL substrate, Bio-Rad) (Hercules, CA, USA) on a ChemiDoc^TM^ MP system. Band intensities were quantified using densitometry and normalized to GAPDH levels using ImageLab software (version 6.1; Bio-Rad). Membranes were stripped using a mild stripping buffer for reuse in subsequent detection.

### 4.4. Histology

Mouse organs were collected, snap-frozen, mounted in O.C.T. (Sakura, Tissue-Tek^®^, Torrance, CA, USA), and stored at −80 °C until sectioning using a Leica CM3050S cryotome (Leica, Wetzlar, Germany). Cryosections of 6 µm were prepared and stored at −80 °C until use. For histological analysis, sections were stained with hematoxylin and eosin (H&E) (Abcam, Cambridge, MA, USA, ab245880) to visualize and quantify cell nuclei and with Masson’s trichrome stain (Abcam, AB150686) to evaluate cytoplasmic content and fibrillar collagen deposition. Slides were imaged using a Keyence BZ−X810 fluorescence microscope (KEYENCE, Osaka, Japan), with both brightfield and darkfield imaging at 10×, 20×, and 40× magnifications.

### 4.5. Immunofluorescence Staining

Immunofluorescence staining was performed on 6 µm cryosections. Tissue sections were fixed in methanol at −20 °C for 10 min, followed by washing in PBS (Sigma Aldrich, 179337). Permeabilization was performed using 0.2% Triton X-100 (Sigma Aldrich; 127K0048) dissolved in PBS for 30 min at room temperature, followed by washing with PBS. The samples were blocked for 1 h in PBS supplemented with 10% fetal bovine serum (FBS, Thermo Fisher Scientific, Whaltham, MA, USA; A5256701). The primary antibody incubation times were optimized for each antibody (Table 1). The secondary antibodies used for detection included Alexa Fluor 555 and Alexa Fluor 488 anti-rabbit and anti-mouse antibodies (Life Technologies, Carlsbad, CA, USA; A31572 anti-rabbit-555, A21202 anti-mouse-488, A31570 anti-mouse-555, and A21206 anti-rabbit-555) (Table 1).

### 4.6. RT-qPCR Analysis

Mouse tissues were frozen in liquid nitrogen and homogenized before mRNA extraction. Homogenization was performed using a glass douncer after initial disruption with a mortar and pestle. Total RNA was extracted using a GeneJET RNA Purification Kit (Thermo Fisher Scientific; K0731) according to the manufacturer’s protocol. The RNA concentration and purity were assessed using a Nanodrop Spectrophotometer ND-1000 (Thermo Fisher Scientific). A High-Capacity cDNA Reverse Transcription Kit (Thermo Fisher Scientific; 4368814) was used to generate cDNA. The resulting cDNA was diluted to a ratio of 1:10 for qPCR analysis and mixed with Power^TM^ Up SYBR^TM^ Green Master Mix (Thermo Fisher Scientific; A25742), primers (Table 2), and nuclease-free water. The thermal cycling program was initiated with a 50 °C equilibration step for 2 min, followed by denaturation at 95 °C for 10 min. This was followed by 50 cycles of denaturation at 95 °C for 15 s and an annealing/extension step at 60 °C for 1 min. The cycling stage was conducted with a melt curve analysis: denaturation at 95 °C for 15 s, annealing at 60 °C, and a final denaturation step at 95 °C for 15 s. GAPDH served as the internal control for normalization. Each experiment was performed in triplicate with three technical replicates for each sample.

### 4.7. Blood Biochemistry and Blood Cytokine Analysis

Mice under isofuran narcosis (5% Isofuran with 100% oxygen flow, 1.5 min exposure) had their retrobulbar vein plexus punctured with a glass capillary (0.8 mm) (5 µL, CAMAG, #022.7729), and blood was collected in lithium heparin-coated sample vessels (LI 1000 A Standard, KABE Labortechnik, Nümbrecht, Germany). We collected 500 µL of total blood from each mouse before centrifugation at 7000× *g* for 15 min to separate the blood plasma at 4 °C. The plasma was then transferred to another heparin-coated vessel and was then snap-frozen in liquid nitrogen.

Blood biochemistry and cytokine levels were measured at the German Mouse Clinic (GMC, Helmholtz center Munich Institute of Experimental Genetics) (Munich, Germany). Blood cytokine levels in some samples were measured for a second time by Dr. Paola Cavalcante (Neurology IV-Neuroimmunology and Neuromuscular Diseases Unit, Fondazione IRCCS Istituto Neurologico Carlo Besta, Milan, Italy). Blood parameters were measured as previously described [78,79].

### 4.8. Electrocardiography and Transthoracic Echocardiography in Awake Mice

Electrocardiography (ECG) and transthoracic echocardiography (TTE) were performed on awake Lmna^G609G/G609G^ mice at 90 days of age. The mice were transported from their housing facility at the German Heart Center, Munich, to the German Mouse Clinic, Helmholtz Center, Munich, for these assessments. For the ECG, mice were acclimatized to the recording platform for 10 min before conscious ECG recordings were acquired using the ECGenie system (Mouse Specifics, Inc., Framingham, MA, USA). For TTE, the body weight was recorded before imaging. Each mouse was placed in a supine position, with the nape supported on the palm of one hand and the tail secured between the last two fingers. A pre-warmed echo transmission gel was applied to the shaved chest area to optimize ultrasound imaging. A Vevo3100 system (VisualSonics, Fujifilm, Toronto, ON, Canada), which is specialized in small-animal echocardiography, was used for image acquisition. Short-axis M-mode images of the left ventricle were obtained at the level of the papillary muscles, which served as anatomical landmarks. A minimum of three M-mode images per mode were captured in each mode. Detailed methods have been previously described by Spielmann et al. 2022 [80].

### 4.9. Statistical Analysis

Histological data analysis was performed using ImageJ software version 1.54k (National Institutes of Health, Bethesda, MD, USA). Data from at least three samples were pooled for statistical analysis. The results are presented as mean ± standard deviation (SD). The statistical significance of any difference was calculated using the one-way ANOVA test with multiple comparisons. Correction for multiple testing was performed using Tukey’s test. The display of significance was shown with the following significance levels: * *p* < 0.05, ** *p* < 0.01; *** *p* < 0.001, **** *p* < 0.0001.

## Figures and Tables

**Table 1:** Antibodies used for immunofluorescence staining.

Antibody ID	Company	Ref #	Incubation Time	Dilution
Lamin A/C	Proteintech (Munich, Germany)	81042-1-RR	ON	1:250
αSMA	MERCK (Darmstadt, Germany)	C6198-100UL	ON	1:1000
Il-6	Invitrogen (Waltham, MA, USA)	P620	ON	1:1000
P16/NK4a	Invitrogen (Waltham, MA, USA)	MA5-17142	ON	1:500
PAI-1/serpine-1	Invitrogen (Waltham, MA, USA)	MA5-17171	ON	1:500
Vimentin	Cell Signaling (Danvers, MA, USA)	D21H3	ON	1:500
Alexa Fluor 555(mouse)	Life Technologies (Carlsbad, CA, USA)	A31570	1 h	1:1000
Alexa Fluor 488(mouse)	Life Technologies (Carlsbad, CA, USA)	A21202	1 h	1:1000
Alexa Fluor 555(rabbit)	Life Technologies (Carlsbad, CA, USA)	A31572	1 h	1:1000
Alexa Fluor 488(rabbit)	Life Technologies (Carlsbad, CA, USA)	A21206	1 h	1:1000

**Table 2:** Forward and reverse primer sequences used for RT-qPCR.

Biomarker	GenID	Forward Primer (5′-3′)	Reverse Primer (5′-3′)
GAPDH	14433	AGGTCGGTGTGAACGGATTTG	TGTAGACCATGTAGTTGAGGTCA
IL-6	16193	TAGTCCTTCCTACCCCAATTTCC	TTGGTCCTTAGCCACTCCTTC
IL-8	20309	CTGGGATTCACCTCAAGAACATC	CAGGGTCAAGGCAAGCCTC
IL-1a	16175	GCACCTTACACCTACCAGAGT	AAACTTCTGCCTGACGAGCTT
IL-1b	16176	GAAATGCCACCTTTTGACAGTG	TGGATGCTCTCATCAGGACAG
IL-10	16153	CTTACTGACTGGCATGAGGATCA	GCAGCTCTAGGAGCATGTGG
TNFα	21926	CCTGTAGCCCACGTCGTAG	GGGAGTAGACAAGGTACAACCC
PAI-1/serpine-1	18787	TTCAGCCCTTGCTTGCCTC	ACACTTTTACTCCGAAGTCGGT
TGF-1b	21803	CTCCCGTGGCTTCTAGTGC	GCCTTAGTTTGGACAGGATCTG
VCAM-1	22329	AGTTGGGGATTCGGTTGTTCT	CCCCTCATTCCTTACCACCC
HIF-1	15251	ACCTTCATCGGAAACTCCAAAG	CTGTTAGGCTGGGAAAAGTTAGG
VEGF	22339	GCACATAGAGAGAATGAGCTTCC	CTCCGCTCTGAACAAGGCT
CXCL1	14825	CTGGGATTCACCTCAAGAACATC	CAGGGTCAAGGCAAGCCTC
CCL2	20296	TTAAAAACCTGGATCGGAACCAA	GCATTAGCTTCAGATTTACGGGT
CTGF	14219	GGGCCTCTTCTGCGATTTC	ATCCAGGCAAGTGCATTGGTA

## Data Availability

Data are contained within the article and Supplementary Materials.

## Supplementary Materials

The following supporting information can be downloaded at https://www.mdpi.com/article/10.3390/ijms26104849/s1.
