# Supplementary material for: Baricitinib and Lonafarnib Synergistically Target Progerin and Inflammation, Improving Lifespan and Health in Progeria Mice
Source: Int J Mol Sci. 2025 May 19;26(10):4849. doi: 10.3390/ijms26104849 (PMC12112389; doi:10.3390/ijms26104849)
Supplement: Supplementary file 1 [file ijms-26-04849-s001.zip › ijms-3612579-supplementary.pdf]

Supplementary Materials

Figure S1

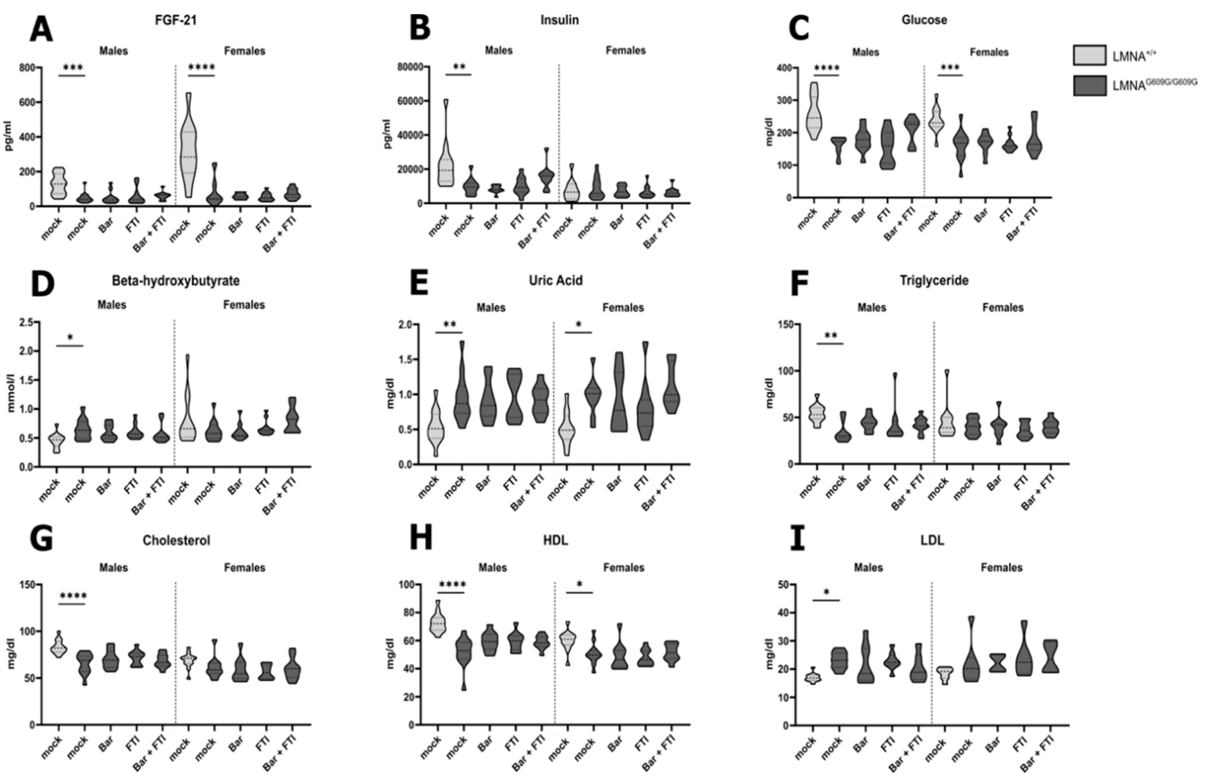

Figure S1: Blood clinical chemistry analysis.
Clinical blood chemistry analysis was performed on all mouse cohorts (mock Lmna+/+ (lightgray), $Lmna^{G609G/G609G}$ mock, $Lmna^{G609G/G609G}$ + BAR, LmnaG609G/G609G + FTI, and $Lmna^{G609G/G609G}$ + BAR + FTI (all LmnaG609G/G609G in dark gray)) with n ranging from 4 to 13. The following parameters were evaluated: (**A**) FGF-21, (**B**) Insulin, (**C**) non-fasting glucose, (**D**) beta-hydroxybutyrate, (**E**) uric acid, (**F**) triglycerides, (**G**) cholesterol, (**H**) HDL and (**I)** LDL. Statistical analysis was carried out using an ordinaryone-way ANOVA test with Tukey's Post Hoc test (* p < 0.05, ** p < 0.01, *** p < 0.001, **** p < 0.0001).

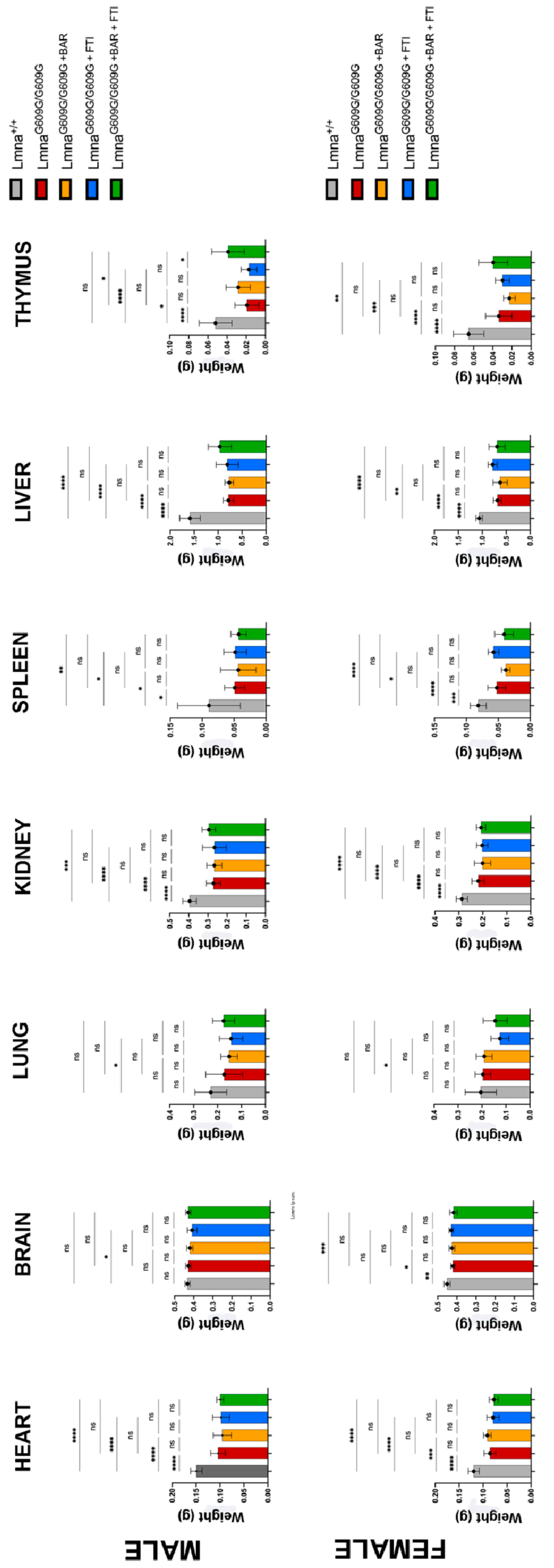

Figure S2: Detailed overview of organ weights from mice at 90 days of age.

The weights of the heart, brain, lungs, kidneys, spleen, liver, and thymus were measured across all five treatment groups: Lmna$^{+/+}$ (gray), Lmna$^{G609G/G609G}$ (red), Lmna$^{G609G/G609G}$ + BAR (orange), Lmna$^{G609G/G609G}$ + FTI (blue), and Lmna$^{G609G/G609G}$ + BAR + FTI (green). Graphs are separated by gender, with male mice shown in the upper row and female mice shown in the lower row (n= 5-10 per group). Statistical significance was determined using an ordinary one-way ANOVA test with Tukey's Post Hoc test. Differences between groups are marked as follows: *p < 0.05; **p < 0.01; ***p < 0.001; ****p < 0.0001.

Figure S3

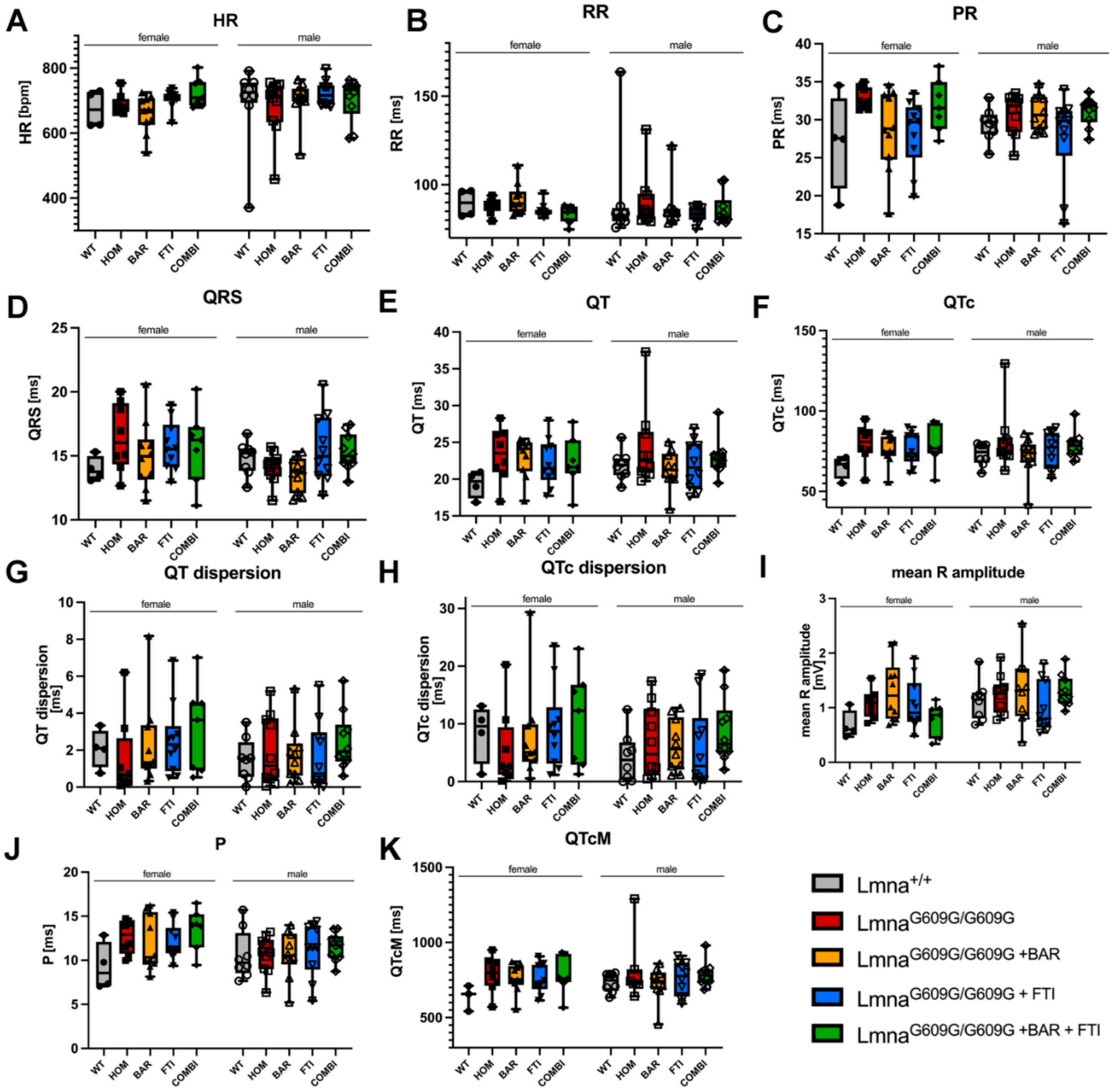

Figure S3: Electrocardiography measurements.
Electrocardiography measurements were conducted on all mouse cohorts (Lmna$^{+/+}$ (gray), Lmna$^{G609G/G609G}$ (red), Lmna$^{G609G/G609G}$ + BAR (orange), Lmna$^{G609G/G609G}$ + FTI (blue), and Lmna$^{G609G/G609G}$ + BAR + FTI (green)) with n ranging between 4 to 10 per group. The following parameters were evaluated: (**A**) heart rate (HR), (**B**) R-R interval, (**C**) P R interval, (**D**) QRS time, (**E**) QT interval, (**F**) corrected QT interval (QTc), (**G**) QT dispersion, (**H**) QTc dispersion, (**I**) mean R amplitude (average amplitude of the R spike), (**J**) P-wave time, and (**K**) median corrected OT time (OTcM).

Figure S4

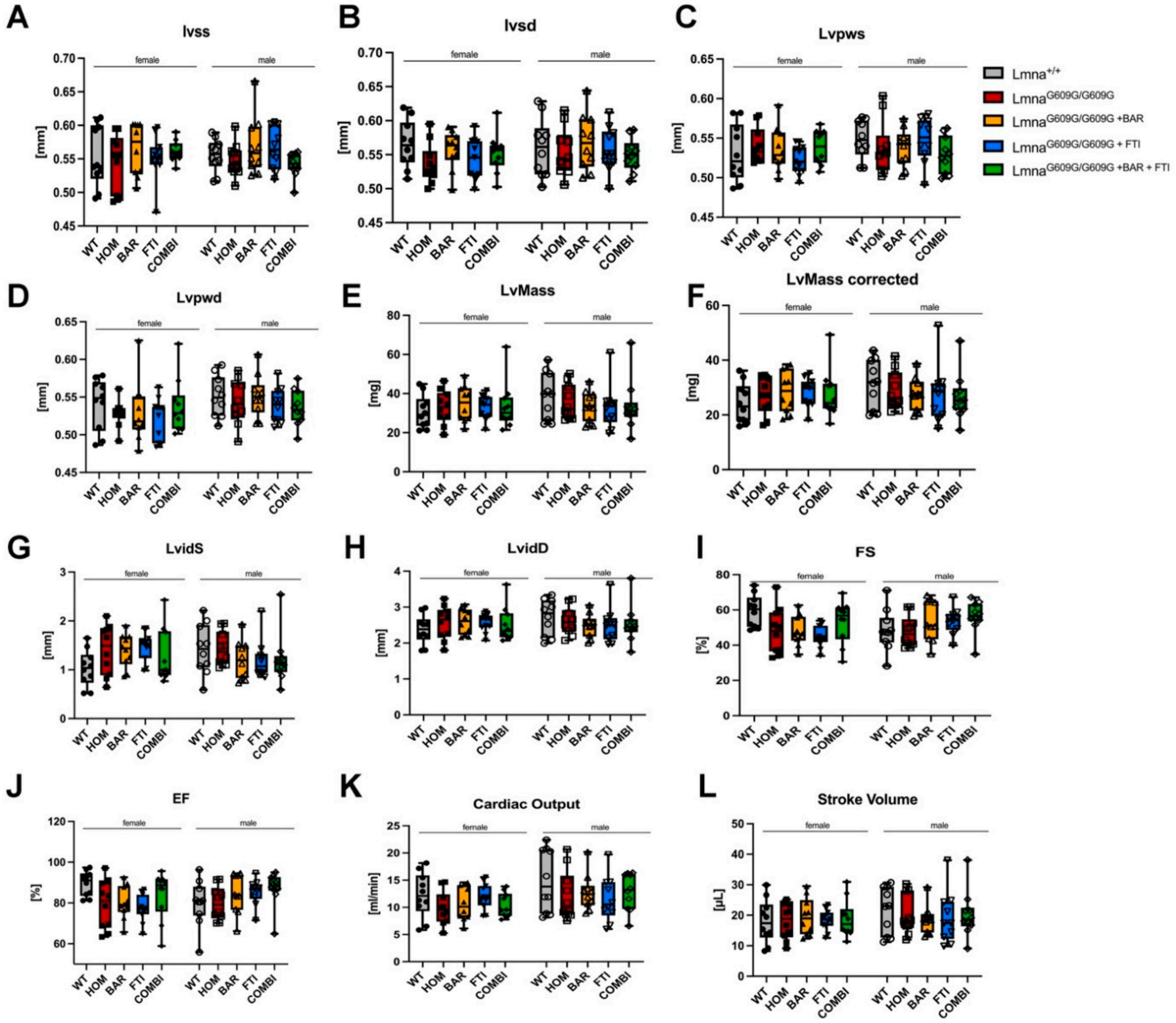

Figure S4: Echocardiographic measurements.

Echocardiographic measurements were conducted on all mouse cohorts (Lmna$^{+/+}$ (grey), Lmna$^{G609G/G609G}$ (red), Lmna$^{G609G/G609G}$ + BAR (orange), Lmna$^{G609G/G609G}$ + FTI (blue), and Lmna$^{G609G/G609G}$ + BAR + FTI (green)) with n = 10 or 11 per group. The following parameters were measured: left ventricle shortening dimension, (**A**) systolic (lvss), (**B**) left ventricle shortening dimension, diastolic (lvsd), (**C**) left ventricular posterior wall thickness, systolic (Lvpws), (**D**) left ventricular posterior wall thickness, diastolic (Lvpwd), (**E**) left ventricular mass (LvMass), (**F**) left ventricular mass corrected (LvMass cor.), (**G**) left ventricular internal diameter systolic (LvidS), (**H**) left ventricular internal diameter diastolic (LvidD), (**I**) fractional shortening (FS), (**J**) ejection fraction (EF), (**K**) Cardiac Output and (**L**) Stroke Volume.

Figure S5

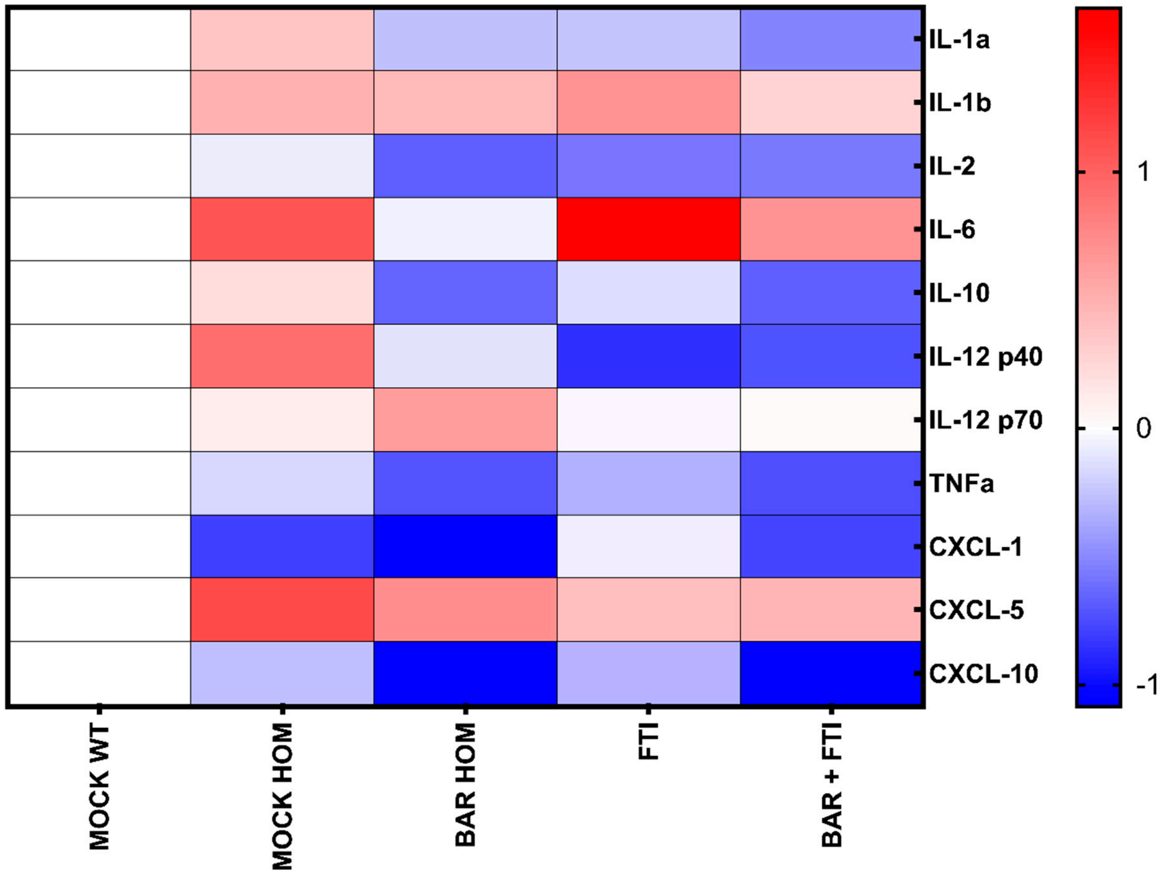

Figure S5: Heat map of blood cytokines.
Blood cytokine levels were analyzed in all treatment cohorts (Lmna$^{+/+}$ (MOCK WT), Lmna$^{G609G/G609G}$ (MOCK HOM), Lmna$^{G609G/G609G}$ + BAR (BAR HOM), Lmna$^{G609G/G609G}$ + FTI (FTI), and Lmna$^{G609G/G609G}$ + BAR + FTI (BAR + FTI)). Each cell represents the fold change in the concentration of individual cytokines relative to the MOCK WT group. We quantified the following markers: IL-1a, IL-1b, Il-2, Il-6, IL-10, IL-12 p40, IL- 12 p70, TNFa, CXCL1, CXCL-5 and CXCL-10. We observed a marked increase in cytokine levels in the Lmna$^{G609G/G609G}$ mock-treated animals, whereas it was reduced in all treatments, showing the most effective reduction in blood cytokine level reductions in the combination-treated animals. The sample sizes ranged from 8 to 25. No sex-related differences were conducted in the analysis. Legend shows the fold change in expression (fold change on a log scale – blue = reduced expression; white = equal expression; red = increased expression).

Figure S6

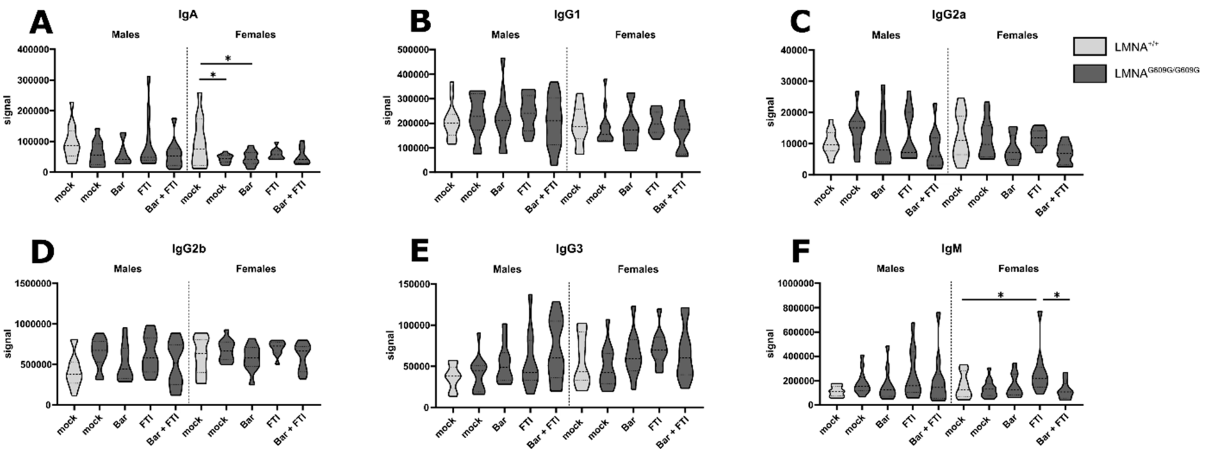

Figure S6: Blood immunoglobulin signals.
Blood immunoglobulin signals were analyzed in all treatment cohorts (mock Lmna$^{+/+}$ (lightgray), Lmna$^{G609G/G609G}$ MOCK, Lmna$^{G609G/G609G}$ + BAR, Lmna$^{G609G/G609G}$ + FTI, and Lmna$^{G609G/G609G}$ + BAR+FTI (all Lmna$^{G609G/G609G}$ are dark gray)). The following markers were quantified: (**A**) IgA, (**B**) IgG1, (**C**) IgG2a, (**D**) IgG2b, (**E**) IgG3 and (**F**) IgM. We observed no clear changes in the biological relevance between the groups. (n = range between 9-13; * p<0,05; ordinary one-way ANOVA test with Tukey´s Post Hoc test.)
